# Confidence measurement in the light of signal detection theory

**DOI:** 10.3389/fpsyg.2014.01455

**Published:** 2014-12-11

**Authors:** Sébastien Massoni, Thibault Gajdos, Jean-Christophe Vergnaud

**Affiliations:** ^1^Queensland Behavioural Economics Group – School of Economics and Finance, Queensland University of TechnologyBrisbane, QLD, Australia; ^2^Groupement de Recherche en Economie Quantitative d’Aix-Marseille, École des Hautes Études en Sciences Sociales, Aix Marseille UniversityMarseille, France; ^3^Centre National de la Recherche ScientifiqueFrance; ^4^Centre d’Economie de la Sorbonne, University of Paris 1Paris, France

**Keywords:** confidence, scoring rules, psychophysics, signal detection theory, incentives, methodology

## Abstract

We compare three alternative methods for eliciting retrospective confidence in the context of a simple perceptual task: the Simple Confidence Rating (a direct report on a numerical scale), the Quadratic Scoring Rule (a post-wagering procedure), and the Matching Probability (MP; a generalization of the no-loss gambling method). We systematically compare the results obtained with these three rules to the theoretical confidence levels that can be inferred from performance in the perceptual task using Signal Detection Theory (SDT). We find that the MP provides better results in that respect. We conclude that MP is particularly well suited for studies of confidence that use SDT as a theoretical framework.

## INTRODUCTION

Humans and animals are able to retrospectively evaluate whether they have or have not made the right decision (e.g., in perceptual, learning, or memory tasks). This metacognitive ability plays an important role in learning and planning future decisions (Dunlosky and Metcalfe, 2008). For instance, humans are not only able to decide whether a visual stimulus did appear or not, but also to say how confident they are in their answer. Such retrospective judgements are often labeled “Type 2 tasks,” as opposed to “Type 1 tasks” which consist of discriminating between perceptual stimuli. In the last few years, considerable progresses had been made in the understanding of behavioral (Smith et al., 2003) and neuronal (Fleming and Dolan, 2012) properties of retrospective confidence. These progressions, rely to a large extent, on the revival of what is known as “Type 2 signal detection analyses” (Clarke et al., 1959; Green and Swets, 1966; Galvin et al., 2003; Maniscalco and Lau, 2012).

Since Green and Swets’s (1966) classical book, Signal Detection Theory (SDT) has been routinely and successfully used in experimental psychology to study simple perceptual decisions. It is postulated that decisions are computed by sensory systems on the basis of noisy signals. The Type 1 task thus reduces to deciding, on the basis of the observation of a signal and some random noise (on some internal axis), whether this observation is due to noise or to the presence of the stimulus.

In the most typical case, it can be shown that the optimal strategy (in the sense of maximizing the likelihood of giving a correct answer) for the subject consists in reporting that the stimulus is present whenever her internal signal is greater than a given threshold. This model thus allows the value of the (non-observable) internal signal used by individuals to make their decisions to be deduced from the observation of their choices.

This reasoning can be pushed one step further to infer confidence, defined as the probability of having made the correct decision (Clarke et al., 1959; Galvin et al., 2003). Indeed, the probability of having made the correct decision can be computed from the value of the perceived signal, through Bayes’ formula. Let us call this model the SDT2 model. A crucial feature of this model is that it allows predictions of retrospective confidence levels based on the observation of Type 1 decisions. This makes it possible, for instance, to measure individuals’ ability to make retrospective confidence judgments by comparing their actual confidence to that predicted by the model (Galvin et al., 2003; Maniscalco and Lau, 2012). Thus, while Type I analyses allow objective performance in perceptual tasks to be assessed, Type II analyses can be used to measure the accuracy of retrospective confidence (see “SDT for Perceptual Tasks” and “SDT for Confidence” below for a more detailed exposition of these models). It is not entirely obvious, however, how confidence judgments should be elicited experimentally. Indeed, experimental studies of consciousness have shown that different methods for eliciting confidence yield different results (Overgaard and Sandberg, 2012)^1^. Thus, the choice of the elicitation method matters. Hence we ask the following question: what is the best way to measure confidence, if we want to measure the sort of confidence described by the SDT2 model? The methods that have been used and discussed in previous studies are of three sorts. The simplest method consists in asking individual to explicitly report their confidence (Simple Confidence Rating, SCR; see Dienes, 2007). A more sophisticated approach, designed to provide individuals incentives to report their “true” confidence, consists in measuring their willingness to bet on the quality of their answer (post-decision wagering procedure; see Persaud et al., 2007). Finally, a third approach consists in measuring individuals’ willingness to trade a gamble based on the correctness of their answer against a lottery with known probabilities (no-loss gambling; see Dienes and Seth, 2010). The aim of this study is to compare confidence judgments elicited with three different rules that belong to each of these categories with the predictions of the SDT2 model.

Interestingly, the data collected with these different methods can be analyzed with the same tools. Using Type 2 SDT, one can for instance quantify the sensitivity of the Type 2 judgments by means of a Type 2 receiver operating characteristics (ROC) curve. The Type 2 ROC curve is obtained by using confidence to classify trials into successes and errors, and then reporting the corresponding hits (i.e., trials correctly classified as a success) and false alarm (i.e., trials erroneously classified as errors) rates. The ROC curve can be used to compute the area under Type 2 ROC curve (AU2ROC), which can be interpreted as follows. Consider a situation in which trials are already correctly classified into two groups (success and failure) and randomly pick a pair of trials, one from each group. The probability that the trial with the higher confidence comes from the success group is equal to the AU2ROC. If an elicitation rule leads individuals to report confidence levels in line with the SDT2 model, then subjects should report confidence levels close to those predicted by SDT. Therefore, the distribution of elicited confidence and the elicited Type 2 ROC should be close to that predicted by SDT. Moreover, elicited Type 2 ROC could never be better than the one predicted, i.e., the elicited AU2ROC should not be greater than the one predicted. Indeed, predicted confidence levels are those of a perfect Bayesian observer, and the subject could therefore not do better (provided the SDT2 model holds, naturally). Furthermore, if a subject is a good (respectively, bad) assessor of her own confidence, then both the distribution of elicited confidence and the Type 2 ROC should be close to (respectively, distant from) the predicted ones. Thus, distances to predicted distribution of confidence and predicted AU2ROC should be positively correlated. Finally, because confidence and the perceptual task are based on the same signals one should observe a positive correlation between performance in the perceptual task and elicited AU2ROC. Studies in humans (Maniscalco and Lau, 2012), rhesus monkeys (Kiani and Shalden, 2009) and rats (Kepecs et al., 2008) indeed found such a relationship, although it has also been shown that, in some circumstances (e.g., subliminal stimuli) Type 1 and Type 2 performances might be disconnected (see, e.g., Kanai et al., 2010; Scott et al., 2014).

We summarize these predictions for future reference. An elicitation rule of confidence in line with the SDT2 model should provide:

– *Prediction 1*: an elicited confidence close to that predicted by SDT2 model;
– *Prediction 2*: an elicited AU2ROC close to the predicted one;
– *Prediction 3*: an elicited AU2ROC not greater than the predicted one;
– *Prediction 4*: the closer the elicited confidence distribution is to the predicted confidence distribution, the closer the elicited AU2ROC is to the predicted one;
– *Prediction 5*: a positive correlation between performance in perceptual task and elicited AU2ROC.

## MATERIALS AND METHODS

### ELICITATION RULES

**FIGURE 1:**
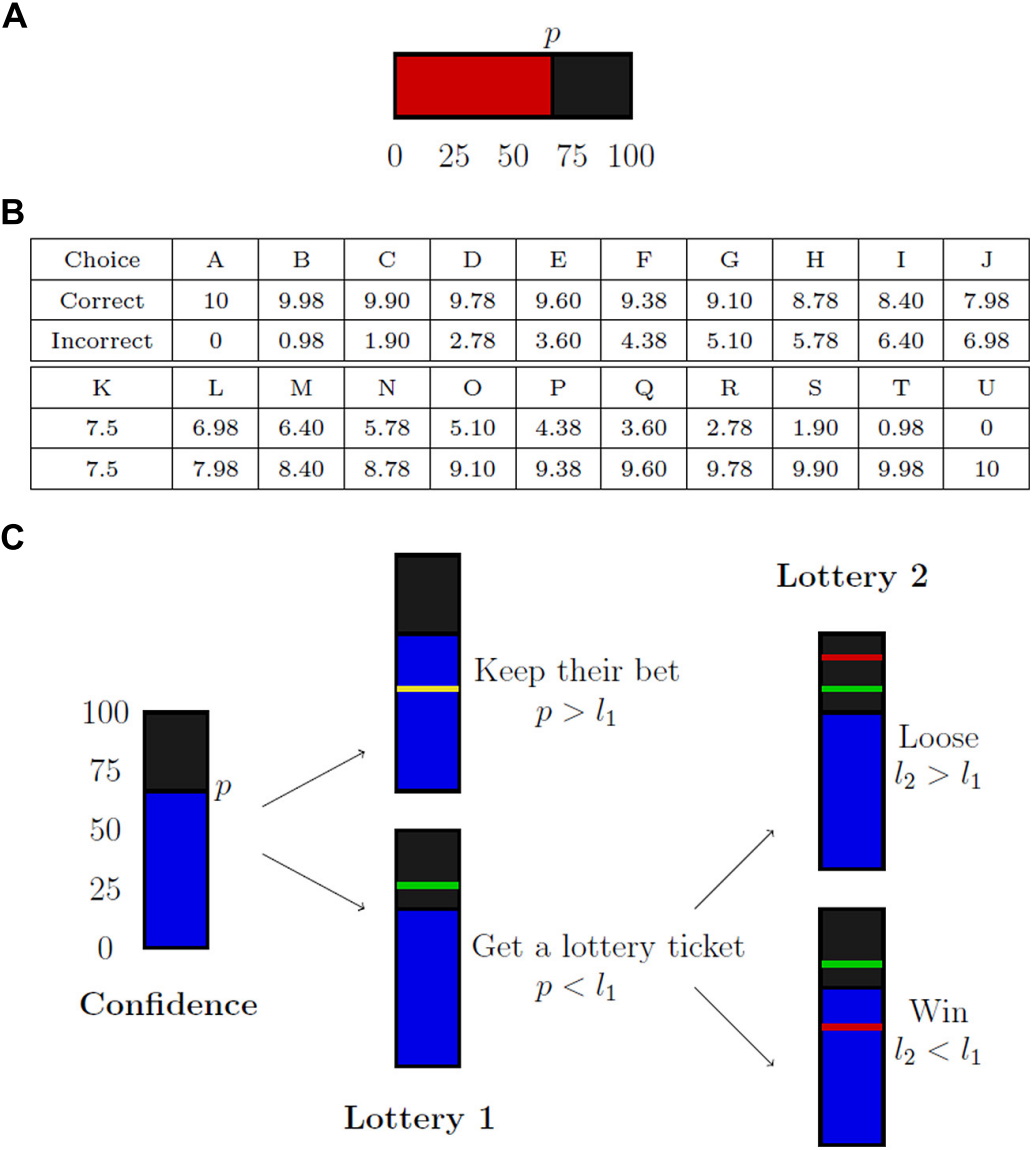
**Elicitation mechanisms for confidence judgments. (A)** Represents the Simple Confidence Rating (SCR), **(B)** the Quadratic Scoring Rule (QSR), and **(C)** the mechanism of the Matching Probability (MP).

The main objective of our experiment is to compare three elicitation rules (see **Figure 1**): the SCR, the Quadratic Scoring Rule (QSR), and the Matching Probability (MP). The SCR is a direct report on a numerical scale. The QSR is a fine-grained version of the post-wagering procedure. The MP is a multi-level extension of the no-loss gambling method proposed by Dienes and Seth (2010). This section is devoted to the presentation of these rules, discussion of their main theoretical properties, and the presentation of their experimental implementation.

#### Simple Confidence Rating

The SCR rule just requires the subject to report her confidence on a numerical scale, without any monetary consequence. Nothing is done to provide incentives. The main advantage of such a rule is of course its simplicity.

We implement the SCR as follows. Subjects have to choose a level of confidence between 0 and 100 (with steps of 5) on a scale (see **Figure 1A**). They are told they are free to use the scale as they want, either by trying to express their confidence level in terms of chance percentages or simply by being consistent in their report with small values for low confidence and high values for high confidence. Payments are independent of elicited probabilities.

#### Quadratic Scoring Rule

The SCR is straightforward and easy to use. Yet, it had been argued that it could yield biased reports, as individuals have no incentive to reveal their “true” confidence, which might require an effort. It has thus been proposed to use an indirect method, called post-decision wagering, based on individual willingness to bet on the quality of their answers (Persaud et al., 2007). For instance, after having made her Type 1 decision, the subject is asked whether she is ready to bet €10, €20, or €50 on being right. The idea is that subjects will choose higher stakes when their confidence is higher. Such a method provides incentives, and can be used for non-humans (Middlebrooks and Sommer, 2011).

The QSR has been introduced in the 1950’s (Brier, 1950) and is extensively used in experimental economics (Nyarko and Schotter, 2002; Palfrey and Wang, 2009) and meteorology (Palmer and Hagedorn, 2006) among others. It is a generalization of the Post-Decision Wagering described above. Assume a subject reports a confidence level equal to *p*. She will then win *a-b(1-p^2^)* € if her answer is accurate, and *a-b (1-(1-p)^2^)* € otherwise, where *a* and *b* are positive constants. The QSR, like the Post-Wagering Method, provides incentives to reveal ones’ true confidence (see Gneiting and Raftery, 2007, for a review of proper scoring rules).

In our experiment, QSR is implemented as follows. We ask subjects to choose among different levels of remunerations (**Figure 1B**). Each letter corresponds to a payment scheme (*x, y*), that yields *x* if their answer is correct and *y* otherwise. These payments are generated using a QSR with parameters *a* = *b* = *10*, and a 0.05 step (i.e., *A* corresponds to *p* = 1, *B* corresponds to *p* = 0.95 and so on). If, for instance, the subject enters *K*, she will obtain a sure payment of 7.5, which is the optimal choice if she maximizes her expected income and believes that she has an equal probability of being correct or not. The unit used for payments are euro cents. Note that there is no explicit reference to probabilities in this procedure. Subjects are not told that payment schemes are linked to confidence levels.

#### Matching probability

Post-decision wagering in general and QSR in particular, could lead to erroneous measures, insofar as variations in confidence measured by such method could to some extent reveal heterogeneity in attitude towards risk or loss, and not in confidence (see Offerman et al., 2009 and Dienes and Seth, 2010). Dienes and Seth (2010) proposed a method, called “no-loss gambling,” that provides incentive and is immune to risk aversion. It consists in asking individuals whether they prefer to be paid according to the correctness of their answer or according to a specified lottery. For instance, individuals are asked whether they prefer to receive €10 in case of success and get nothing otherwise, or to toss a coin and receive €10 if it turns Heads and nothing otherwise. The idea here is that if a subject prefers to be paid according to the correctness of her answer, it indicates that her confidence in her decision is higher than 50%.

The matching probability (MP) is a variant of the Becker-DeGroot-Marshak mechanism (Becker et al., 1964), and generalizes the no loss gambling method proposed by Dienes and Seth (2010). To elicit a subject’s subjective probability about an event *E*, the subject is asked to provide the probability *p* that makes her indifferent between a lottery *L(E)* that gives a positive reward *x* if *E* happens, and 0 otherwise and a lottery *L(p)* that yields a positive reward *x* with probability *p*, and 0 with probability *(1–p)*. A random number *q* is then drawn in the interval [0,1]. If *q* is smaller than *p*, the subject is paid according to the lottery *L(E)*. Otherwise, the subject is paid according to a lottery *L(q)* that yields *x* with probability *q* and 0 with probability *(1–q)*.

The no-loss gambling method proposed by Dienes and Seth (2010) is a particular case of the MP. Dienes and Seth (2010) were interested in deciding whether subject’s confidence is equal to, or higher than, 50%. The method they propose essentially works as follows. If the subject provides a probability higher than 50%, she is paid according to her answer within the Type 1 task. If she reports a probability equal or smaller than 50%, she is paid according to a 50–50 lottery. This corresponds exactly to the MP under the following two conditions: (i) the subject can only report two confidence levels: “low” (i.e., 50% or below) or “high” (i.e., higher than 50%), and (ii) q is fixed at 0.5 such that the lottery *L*(*q*) has a 50% chance of reward. In the general case, *q* needs to be random to prevent subjects overstating their confidence. This is not needed in the no-loss gambling method as it only allows binary answers.

The MP procedure provides incentives for subjects to reveal their subjective probability truthfully. To make this clear, suppose that the subject thinks her probability of success is *p* but reports a probability *r ≠ p*. First consider the case where *r < p*. The lotteries according to which the subject (given her subjective probability *p*) is paid are represented in the following **Table 1**, as a function of the random value *q*.

**Table 1:** Incentives of the MP against an unde-report of confidence.

	*q* < *r* < *p*	*r* < *q* < *p*	*r* < *p* < *q*
Reports *r* < *p*	*L*(*p*)	*L*(*q*)	*L*(*q*)
Reports *p*	*L*(*p*)	*L*(*p*)	*L*(*q*)

Note that whenever *r < q < p*, *L(p)* yields a higher payment than *L(q)*. Thus, the subject’s expected payoff is higher when reporting *p* than when reporting *r < p.*

Similarly, assume that the subject reports *r > p*. Her payments (according to her subjective probability *p*) are described in the following **Table 2**.

**Table 2:** Incentives of the MP against an over-report of confidence.

	*q* < *p* < *r*	*p* < *q* < *r*	*p* < *r* < *q*
Reports *r* > *p*	*L*(*p*)	*L*(*p*)	*L*(*q*)
Reports *p*	*L*(*p*)	*L*(*q*)	*L*(*q*)

Note that whenever *p < q < r*, *L(q)* yields a higher payment than *L(p)*. Thus, the subject’s expected payoff is higher when reporting *p* than when reporting *r > p.*

A major advantage of the MP is that it provides the subject incentives to reveal her subjective probabilities truthfully, while not being contaminated by her attitude towards risk (see Dienes and Seth, 2010). The MP mechanism might seem complicated. It is thus of interest to investigate whether individuals are able to efficiently use it. As we will see, such is actually the case.

In practice the MP is implemented using a scale of 0–100, with steps of 5 (see **Figure 1C**). After having completed the perceptual task, subjects are told that they are entitled to a ticket for a lottery based on their answers’ accuracy. This lottery gives them €0.10 if their answer is correct, and 0 otherwise. Subjects have then to report on a scale ranging from 0–100 the minimal percentage of chance *p* they require to accept in an exchange between their lottery ticket and a lottery ticket that gives *p* chances of winning €0.10. A number *l_1_* is drawn according to a uniform distribution between 40 and 100. If *l_1_* is smaller than *p*, subjects keep their initial lottery ticket. If *l_1_* is higher than *p*, they are paid according to a lottery that gives them *l_1_* chances of winning. In this case, a random draw determines the payment: a number *l_2_* is determined using a uniform distribution between 0 and 100, the lottery leads to a win if *l_1_* is higher than *l_2_*.

### EXPERIMENTAL PROCEDURE

#### Participants

The experiment took place in June and October 2009 at the Laboratory of Experimental Economics in Paris (LEEP). One hundred and thirteen subjects were recruited using ORSEE (Online Recruitment System for Experimental Economics) and the LEEP’s database. They were students from all fields. Participants were tested in groups of 15–20 at once. We ran two sessions for each rule, resulting in 35–40 subjects for each rule^2^. In each session, subjects were randomly affected to a workstation and they answer anonymously to several demographic questions (age, sex, domain of study) at the beginning of the experiment. There was no matching between the data collected anonymously during the experiment and the information contained in the LEEP’s database. We received from the LEEP only the experimental data. Because no nominative information was collected and no medical or physical act was involved, under French regulatory ethics no approval by an IRB was needed^3^. We just asked subjects to provide a written informed consent. Subjects were informed that the experiment was fully anonymous and will be only used for the purposes of scientific research. The experiments last for about 90 min and they were paid on a performance basis (€19 on average).

#### Stimuli

This computer-based experiment uses MATLAB with the Psychophysics Toolbox version 3 (Brainard, 1997) and was conducted on computers with 1024 × 768 screens.

**FIGURE 2:**
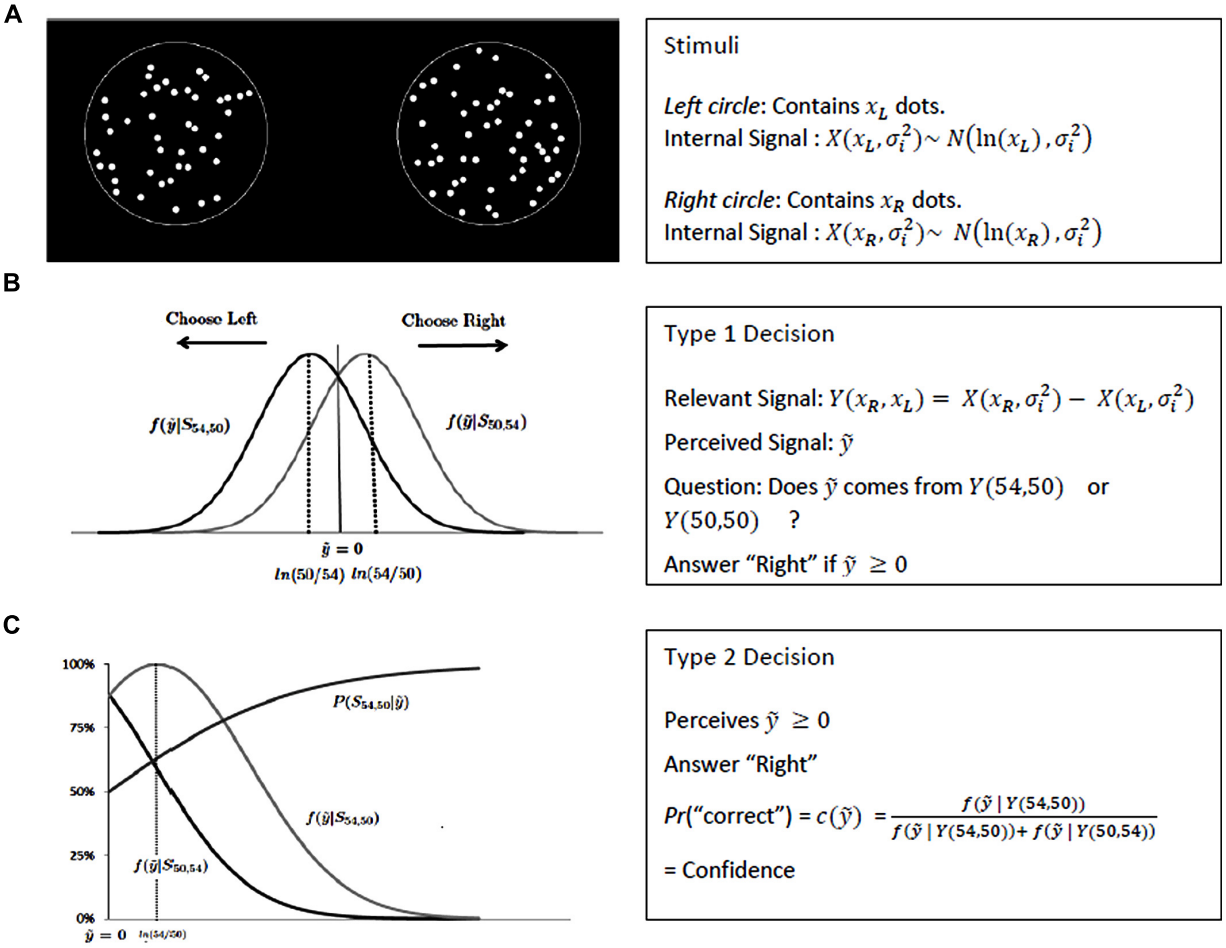
**SDT framework. (A)** Presents an example of stimuli used for the task and details how the visual signal are coded by SDT. **(B)** Defines the optimal criterion of the type 1 decision, while **(C)** extends SDT to type 2 decision with the computation of confidence.

The perceptual task we use is a two-alternative forced choice, which is known to be a convenient paradigm for SDT analysis (see, e.g., Bogacz et al., 2006). Subjects have to compare the number of dots contained in two circles (see **Figure 2A**).

First two outline circles are displayed with fixation crosses at their center. The subjects initiate the trial by pressing the “space” key on standard computer keyboard. The two circles (diameter 5.1°) appears immediately at eccentricities of ±8.9° with variable number of dots (diameter 0.4°) displayed inside the circles for 700 ms overall. Subjects have to tell which circle contains the higher number of dots without a time limit. They are asked to respond left or right by pressing the “f” or “j” keys, respectively. We allow the difficulty of the task to vary, by changing the spread of the number of dots between the two circles. One of the two circles always contains 50 dots. Its position (to the left or the right of the screen) is randomly chosen for each trial. The other circle contains 50 ± α_j_ dots, where α_j_ is randomly chosen for each trial in the set {α_0_, α_1_, α_2_, α_3_, α_4_}; for all subjects, α_0_ = 0 and α_4_ = 25. The intermediate difficulty levels are adapted to each participant, in order to control for differences in individual abilities. During a training part of the experiment, α_2_ is adjusted so that the subject succeeds in 71% of the cases at that level of difficulty. This calibration is done by using a one-up two-down psychophysics staircase with 30 reversals (Levitt, 1971). The two other parameters α_1_ and α_2_ are then given by α_3_ = 2.α_2_ and α_1_ = α_2_/2 if α_2_ is even, and α_1_ = (α_2_ + 1)/2 if α_2_ is odd. The subjects are aware that there is an equal probability for each circle to be the one with the largest number of dots.

This task was completed by a quiz with questions related to logic and general knowledge that is not used in the present study.

#### Procedure

In a given experimental session, a single elicitation rule (the same for all subjects) is used. Thus, our study is based on a between-subjects analysis with a simple 3 × 1 design^4^. After presentation of the instructions (that include a detailed presentation of the elicitation rule with explanations of the underlying mechanisms and various examples of ratings strategies^5^) and a short questionnaire, the experiment is divided in three parts.

In the first part of the experiment, subjects have to answer a randomly chosen quiz (logic or general knowledge) and to provide their confidence for each answer. They are given no feedback on their answers.

During the second part of the experiment, subjects have to perform the perceptual task. They begin with a training phase during which the difficulty of the task is calibrated. Confidence is not elicited during this first phase, and they get feedback on their success after each trial. Subjects then perform overall 100 trials of the perceptual task (20 trials by difficulty levels with randomized order), and provide their confidence in their answer for each trial. Both answers are done without a time constraint. They get feedback on their success in the task and the accuracy of their reported confidence. Furthermore, for every 10 trials, subjects receive a summary of their performance in the last ten trials in terms of success rate and cumulated gains.

The last part of the experiment is similar to the first one, except that subjects have to answer the quiz that has not been selected in the first part.

At the end of the experiment, participants receive their payments. There is a show-up fee of €5. Subjects are paid for each trial. For groups using the QSR or the matching probability, each 100 trials of the perceptual task is rewarded according to the elicitation rule used, with a maximum payment of €0.10 and a minimum of €0. Subjects in the group using the SCR are paid €0.10 for each correct answer. Subjects are also paid for the quiz task, but this payment is completely independent.

### ANALYSIS

#### SDT for perceptual tasks

Since Green and Swets’s (1966) classical book, SDT has been routinely and successfully used in experimental psychology to study individual decisions in perceptual tasks. Let us apply it to simple perception we used in our experiment (see Stimuli). The two circles are only displayed for a short fraction of time, 700 ms, so that it is not possible to count the dots. However, the subject is aware that a circle can only contain 54 or 50 dots, and that there is an equal probability for each circle to be the one with the largest number of dots.

It is postulated that stimuli are perceived as noisy signals by the sensory system. Here, we are interested in the numerosity of the circles, i.e., the number of dots they contain. It is assumed that, when presented with a circle that contains *x* dots, the sensory system actually observes the realization $\overset{\Lambda }{x}$ of a random signal *X(x,* σ_i_) that is distributed according to a Gaussian law, with mean ln(*x*) and variance σ_i_^2^, where σ_i_ is a parameter describing the degree of precision of the internal representation of numerosity in the brain (see Nieder and Miller (2004), Piazza et al. (2004), Pica et al. (2004), and Nieder (2005) for behavioral, neurophysiological, and neuroimaging justifications of this model, and in particular for the use of a logarithmic scale). When observing two circles with respectively *x_L_* and *x_R_* dots (where *L* and *R* stand for left and right, respectively), the subject thus actually observes the realizations $\overset{\Lambda }{x}$_R_ and $\overset{\Lambda }{x}$_L_ of two random signals *X(x_R_,* σ_i_) and *X(x_L_,* σ_i_) (see **Figure 2A**). Because the subject has to decide which circle contains the largest number of dots, the relevant information is actually the *difference* between the two signals. We thus assume that, when presented with the circles and asked which one contains the largest number of dots, the subject’s decision is based on a noisy signal *Y(x_R_,x_L_)* = *X(x_R_,* σ_i_) – *X(x_L_,* σ_i_) (*Y* thus also depends on σ_i_, but we omit this variable for notational simplicity). For a given trial, the subject thus perceives a signal ŷ and has to decide whether it has been generated by *Y*(54,50; i.e., there are 54 dots in the right circle, and 50 in the left one), or by *Y*(50,54; i.e., there are 50 dots in the right circle, and 54 in the left one). Let f(y|x_R′_x_L_) be the density function of *Y* conditional to *X(x_R_,* σ_i_) and *X(x_L_,* σ_i_). Since she is aware that there is an equal chance for any circle to be the one containing the largest number of dots, her optimal strategy (in the sense of Neyman-Pearson) is based on the likelihood ratio and consists in answering “Right” whenever ŷ ≥ 0, and “Left” otherwise (see **Figure 2B**; Green and Swets, 1966).

#### SDT for confidence

The Bayesian reasoning can be pushed further (see Galvin et al., 2003; Fleming and Dolan, 2010; Rounis et al., 2010; Maniscalco and Lau, 2012; Barett et al., 2013) to model how subjects make confidence judgments in terms of probabilities about their decisions in a perceptual task. Such judgments are known as “Type 2 tasks” (Clarke et al., 1959; Pollack, 1959), as opposed to “Type 1 tasks” which consist of discriminating between perceptual stimuli. Consider a trial where the subject perceives a positive signal ŷ, and therefore answers “Right.” Based on the SDT model presented above, one can easily deduce the probability that she gives the correct answer. According to the Bayes rule, it is equal to P(Y (54,50)|ŷ) = $\frac{f\left(\overset{\Lambda }{y}|Y\left(54,50\right)\right)}{f(\overset{\Lambda }{y}|Y\left(54,50\right))+f\left(\overset{\Lambda }{y}|Y\left(50,54\right)\right)}$ (see **Figure 2C**). This confidence based on signal detection will be called SD-confidence (where “SD” stands for “Signal Detection”) in the sequel.

**FIGURE 3:**
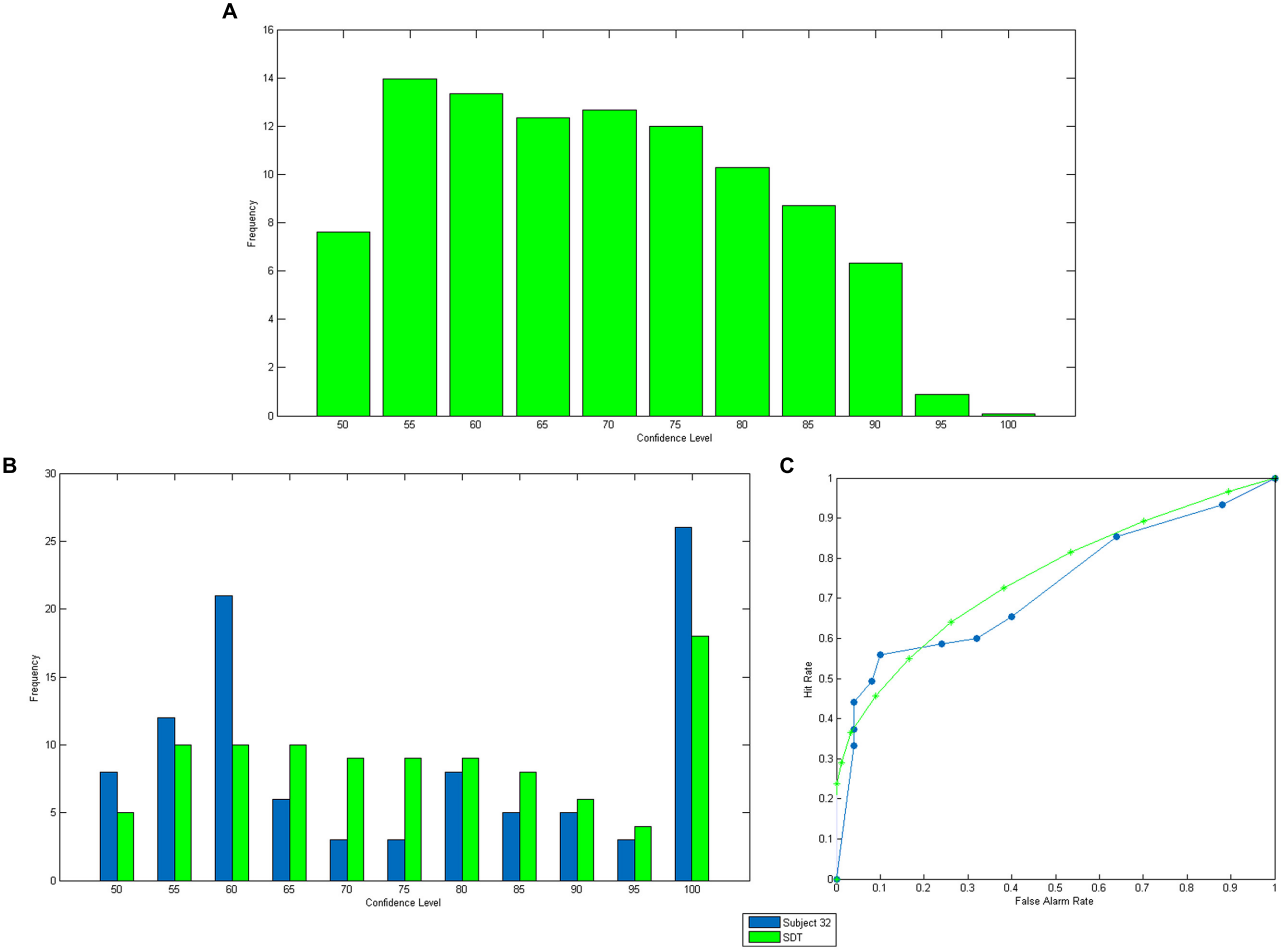
**Predictions of confidence. (A)** is the predicted distribution of SD-confidence. **(B,C)** are respectively the observed and predicted confidence distribution and AU2ROC for a specific subject.

Since we control for the difficulty levels of the stimuli used in the perceptual task, we can use SDT to estimate subjects’ perceptual sensitivity from behavioral data (i.e., using success rates). This leads to an estimation of the distribution of the internal signal used by the subject when performing the perceptual task. With this in hand, the SDT model provides precise predictions about the SD-confidence levels of an ideal (i.e., optimal and Bayesian) observer who receives the same internal signals as the subject. First we can compute the *distribution* of SD-confidence. Indeed, SDT predicts the SD-confidence level associated to each level of the internal signal (**Figure 2C**). It also provides the probability to reach any confidence level. Given a probability *p*, ŷ_p_ is such that P(Y (54,50)|ŷ_p_) = p. The probability of observing a confidence level above *p* is $\int_{{\overset{\Lambda }{y}}_{p}}^{\infty }\left(0.5.f(\overset{\Lambda }{y}|Y\left(54,50\right))+0.5.f\left(\overset{\Lambda }{y}|Y\left(50,54\right)\right)\right)d\overset{\Lambda }{y}$. In our experiment where the confidence scale is discrete with 5% increments, we can thus deduce the probability distribution of SD-confidence (**Figure 3A**).

One drawback of the distribution of SD-confidence is that it does not keep track of the trial-by-trial relationship between SD-confidence and success in the perceptual task. This link can be represented by a ROC curve (Green and Swets, 1966). Consider a given level of SD-confidence, say 70%. Assume that one uses this confidence level to decide whether the answer was correct or not. Thus, all trials for which the SD-confidence is higher than 70% will be classified as correct, whereas the others will be classified as incorrect. This classification is of course imperfect. But we can compute the false alarm rate (i.e., the proportion of trials that would be wrongly classified as correct) and the hit rate (i.e., the proportion of trials that would be correctly classified as correct). Thus, for each SD-confidence level, we can associate a point on a graph with hit rates on the vertical axis, and false alarm rates on the horizontal axis. The curve that relates all the points obtained by varying the SD-confidence level is the type 2 ROC curve. To measure how accurate confidence is predictive of success, one usually computes the area under this ROC curve (AU2ROC) which has the following statistical meaning. Consider a situation in which trials are already correctly classified into two groups (success and failure) and randomly pick a pair of trials, one from each group. The probability that the trial with the higher confidence comes from the success group is equal to the AU2ROC.

The above analysis can be readily extended to the case where the task difficulty varies across trials, as is the case in our experiment, by assuming that the ideal observer has correct priors about the distribution of difficulty levels. To illustrate this method, we computed the distribution of elicited confidence and predicted SD-confidence (**Figure 3B**) for a subject in our experiment. It can be observed that the data fits SDT predictions nicely. We also computed, for the same subject, the observed and predicted Type 2 ROC curve (**Figure 3C**). The predicted AU2ROC is equal to 0.75, which is very close to the observed AU2ROC (equal to 0.72). Note that the shape of confidence distribution for this subject differs from that shown in **Figure 3A**. This is due to the fact that **Figure 3A** has been drawn under the assumption of a unique difficulty level, while predicted SD confidence and Type 2 ROC in **Figure 3** have been computed using the actual distribution of difficulty levels in our experiment.

#### Statistical tools

The relationships between different measures were analyzed with Pearson’s product-moment correlations. Comparisons of their means were conducted using paired *t*-tests. Multiple comparisons of means were based on *post hoc* tests after one-way ANOVA. We used Fisher-Hayter pairwise comparison as this method handles well unequal cell sizes and provides a powerful test based on studentized range distribution. Comparisons of correlations were based on Fisher’s *z* transformation tests. We use two goodness-of-fit tests: the Chi-Square test, that measures the distance between empirical and predicted density functions, the Kolmogorov–Smirnov test, which measures the distance between empirical and predicted cumulative distribution functions (see Zar, 2010).

Finally, in order to measure the distances between observed and predicted distributions of confidence we construct a measure, called below *ROC_distance*, as follows:

$$R\,O\,C\,\;\;\;d\,i\,s\,\tan c\,e=\frac{\left|\mathrm{Pr}e\,d\,i\,c\,t\,e\,d\,\;\;A\,U\,2\,R\,O\,C-O\,b\,s\,e\,r\,v\,e\,d\,\;\;A\,U\,2\,R\,O\,C\;\right|}{\mathrm{Pr}e\,d\,i\,c\,t\,e\,d\,\;\;A\,U\,2\,R\,O\,C-0.5}$$

This measure captures the percentage of change of observed metacognitive performances relative to the predicted one. We use it to measure the relationship between the distances of confidence distributions and AU2ROC for observed and predicted confidence.

## RESULTS

### ELICITED CONFIDENCE: DESCRIPTIVE ANALYSIS

**FIGURE 4:**
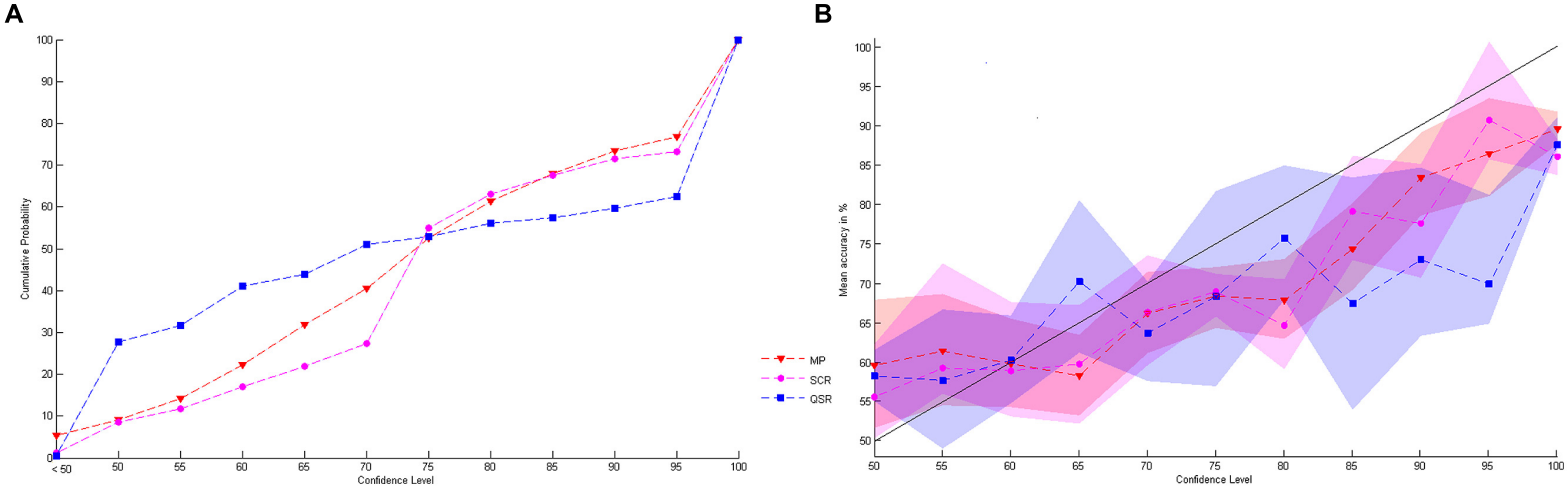
**Stated values of confidence. (A)** Shows the cumulative probability distribution of stated confidence levels for the three rules. **(B)** Represents the link between stated confidence and mean level of accuracy for the three rules with 95% confidence intervals.

We start by presenting some basic facts concerning elicited confidence. First, we observe that while the cumulative distributions of elicited confidence obtained by the SCR and the MP are similar, the one corresponding to the QSR differs substantially (see **Figure 4A**). The difference is mainly due to the fact that the confidence levels elicited by the QSR are strongly concentrated on two values, 50 and 100%. Almost two thirds of elicited probabilities are either equal to 50% (25.5%) or 100% (39.8%) when the QSR is used, which is twice as much as for the two other rules (MP: 3.8% on 50% and 22.6% on 100%: SCR: 7.5% on 50% and 25.0% on 100%).

Let us next examine how the subjects’ stated confidence is related to their actual success rate (see **Figure 4B**). An initial observation is that, whatever the elicitation rule used, subjects are generally overconfident. Moreover, the difference between stated confidence and observed success rates increases with stated confidence. If we consider all trials for which subjects reported a 100% probability of success, we observe an actual success rate of 86.9% only. On the other hand, low confidence levels (50%) correspond to actual success rates that are slightly higher than 50% (57.8%). In terms of guessing criterion only the QSR provides a significant performance above baseline while reporting 50% of confidence^6^ (mean 0.5889, SD 0.13, *t*(30) = 3.5675, *P* = 0.0013 while the MP gives a mean 0.5668, SD 0.29, *t*(29) = 1.2265, *P* = 0.2302 and the SCR a mean 0.5325, SD 0.30, *t*(22) = 0.4932, *P* = 0.6270). But we cannot find any significant differences between the mean performances at 50% of confidence for the three rules (One-way ANOVA: *F*(2,81) = 0.32, *P* = 0.7285). Finally, we note that none of the elicitation rules provides a strictly increasing relationship between stated confidence and the actual success rate.

The QSR and the MP are cognitively demanding and we expect their performances to increase with practice. Our experiment is designed so as to offer subjects the opportunity of learning by using feedback. During the second part of the experiment, the subjects used the elicitation rule with feedback a 100 times. Thus they could have learnt to use the elicitation rule during this stage. We can therefore measure learning effects by comparing results for the first half (first 50 trials) and the second half (50 last trials) of the perceptual task. Overall we observe a learning effect for discrimination ability: the AU2ROC is systematically higher in the second part (mean 0.6573, SD 0.09) than in the first part (mean 0.6729, SD 0.09, *t*(113) = -1.8472, *P* = 0.0337). Nevertheless this learning effect is too weak to be observed at the rule level (for MP: *t*(40) = 0.8814, *P* = 0.1918; for QSR: *t*(35) = 1.3079, *P* = 0.0998; and for SCR: *t*(38) = 0.9935, *P* = 0.1635). Since the increase is quite similar for the three rules, it is possible that this learning effect reflects more of an increase in metacognitive abilities than an increase in the understanding of the QSR and the MP.

Finally we can check whether our paradigm is validated by the data. We observe that the range of value of α_2_ (i.e., the number of dots leading to 71% of accuracy according to the staircase procedure) goes from 1 to 7 with the following distribution among subjects: 3 subjects with 1 dot of difference, 29 subjects with 2, 49 subjects with 3, 21 subjects with 4, 9 subjects with 5, 1 subject with 6, and 1 subject with 7. As expected the mean performances follow the level of difficulty with a performance not statistically significant different from the chance for α_0_ i.e. no difference of dots between circles (mean 0.5042 SD 0.50, *t*(113) = 1.8378, *P* = 0.0687); an almost perfect accuracy for α_4_, i.e., 25 dots of difference (mean 0.9974 SD 0.05); and an increase performance for respectively α_1_ = α_2_/2, α_2_ and α_3_ = 2.α_2_ (α_1_: mean 0.5971 SD 0.49; α_2_: mean 0.6758 SD 0.46; α_3_: mean 0.8050 SD 0.39)

### ELICITED CONFIDENCE AND SDT PREDICTIONS

We now consider to what extent elicitation rules lead individuals to report confidence levels that are close to those predicted by the SDT2 model. The first thing we need is to compute predicted confidence in the perceptual task.

**FIGURE 5:**
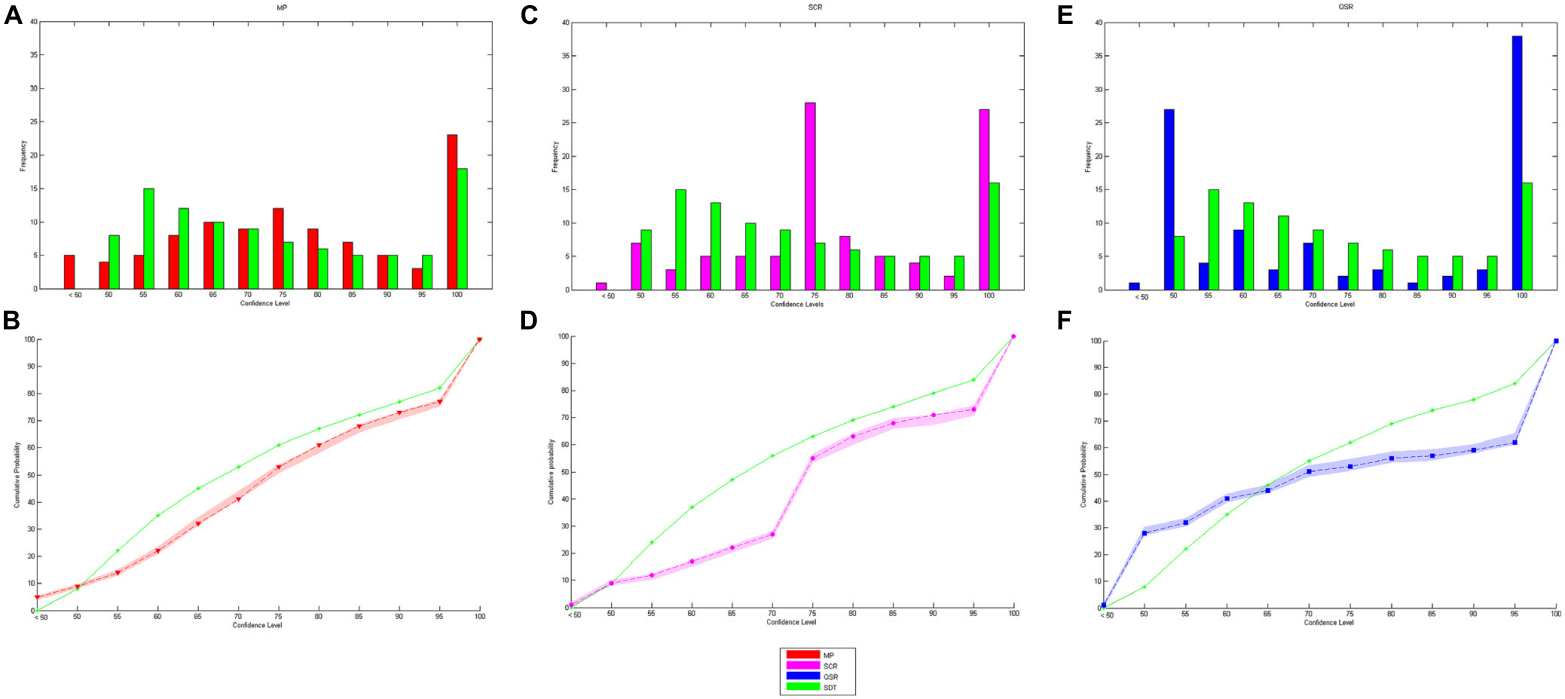
**Observed and predicted distribution and cumulative distribution of confidence. (A,C,E)** are respectively the observed and predicted distribution of confidence for MP, SCR, and QSR while **(B,D,F)** are the cumulative distribution of confidence for MP, SCR, and QSR.

We proceed by examining in turn each of the predictions 1–5 listed at the end of the introduction. Let us start with prediction 1, which states that elicited confidence should be close to predicted confidence. An initial answer is provided by comparing elicited confidence and predicted confidence distributions. **Figure 5** reports the elicited confidence and predicted confidence distributions and cumulative distribution for each elicitation rule (data is pooled across all levels of difficulty and all subjects). The MP seems to be the rule that leads to the best fit. The SCR is plagued by the large proportion of elicited confidence levels equal to 75%, which is the pre-filled value of the scale^7^. Confidence levels elicited with the QSR are those that differs the most from predicted confidence. There is a peak at a 50% confidence level, which is expected because of risk aversion. But we also observe a high peak at the 100% value (with 39.9% of the answers), which cannot be explained by risk aversion, and which does not correspond to predictions of SDT (only 18% of the answer should take this value according to SDT).

**FIGURE 6:**
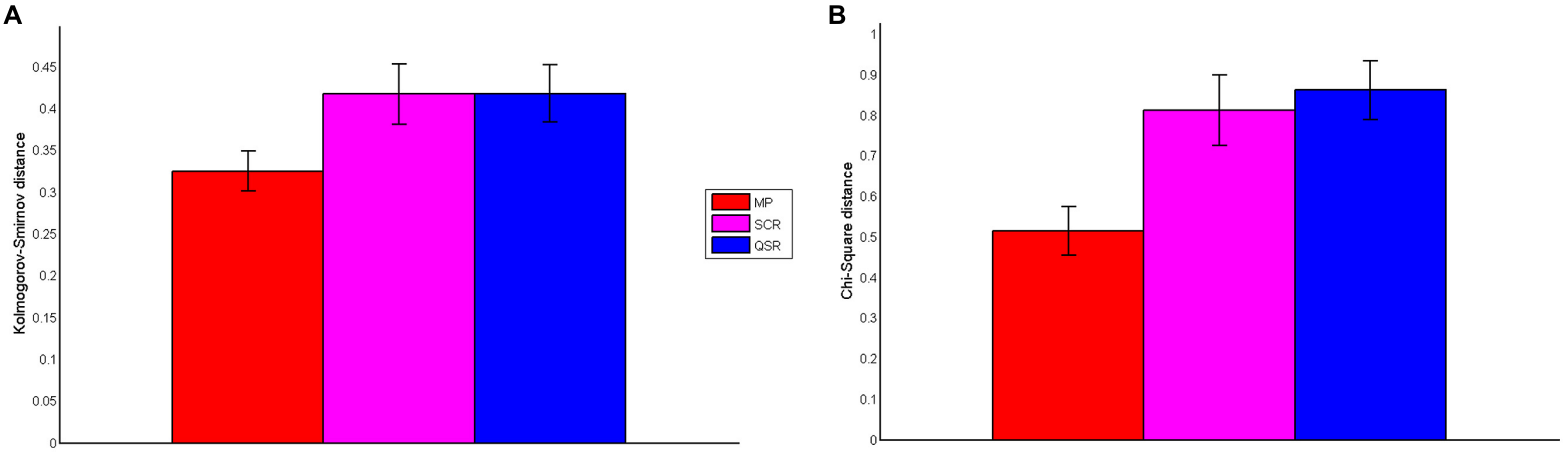
**Distance between confidence distributions. (A)** Presents the Kolmogorov–Smirnov distance between the cumulative distribution of stated and predicted confidence for the three rules. **(B)** Shows the Chi-Square distance between observed and predicted confidence distribution of the three rules.

To confirm the visual impression that MP leads to the best fit between elicited confidence and predicted confidence, we computed the Chi-Square distance between the elicited confidence and predicted confidence distributions, and the Kolmogorov–Smirnov (KS) distance between the elicited confidence and predicted confidence cumulative distributions. We report the two distances for the three rules (with SD in brackets) in **Figure 6**. One-way ANOVA shows that there is a difference of distances between group: Chi-Square distance: *F*(2,113) = 6.98, *P* = 0.0014): KS distance: *F*(2,113) = 3.13, *P* = 0.0476). Furthermore Fisher-Hayter *post hoc* tests show that the two distances are significantly lower for the MP (Chi-Square distance: mean 0.5152, SD 0.37; KS distance: mean 0.3252, SD 0.15) than for the QSR (Chi-Square distance: mean 0.8621, SD 0.42, FH-test(73) = –4.8182, *P* = 0.0001; KS distance: mean 0.4182, SD 0.20, FH-test(73) = –3.0158, *P* = 0.04) and the SCR (Chi-Square distance: mean 0.8129, SD 0.52, FH-test(75) = –4.1952, *P* = 0.003; the KS distance: mean 0.4158, SD 0.22, FH-test(75) = –3.0659, *P* = 0.04) while there are no significant differences between QSR and SCR (Chi-Square distance: FH-test(70) = 0.6713, *P* = 0.36; KS distance: FH-test(71) = 0.0150, *P* = 1.000). We also found that the two distances are strongly correlated (*r* = 0.85, *P* < 0.00001).

**FIGURE 7:**
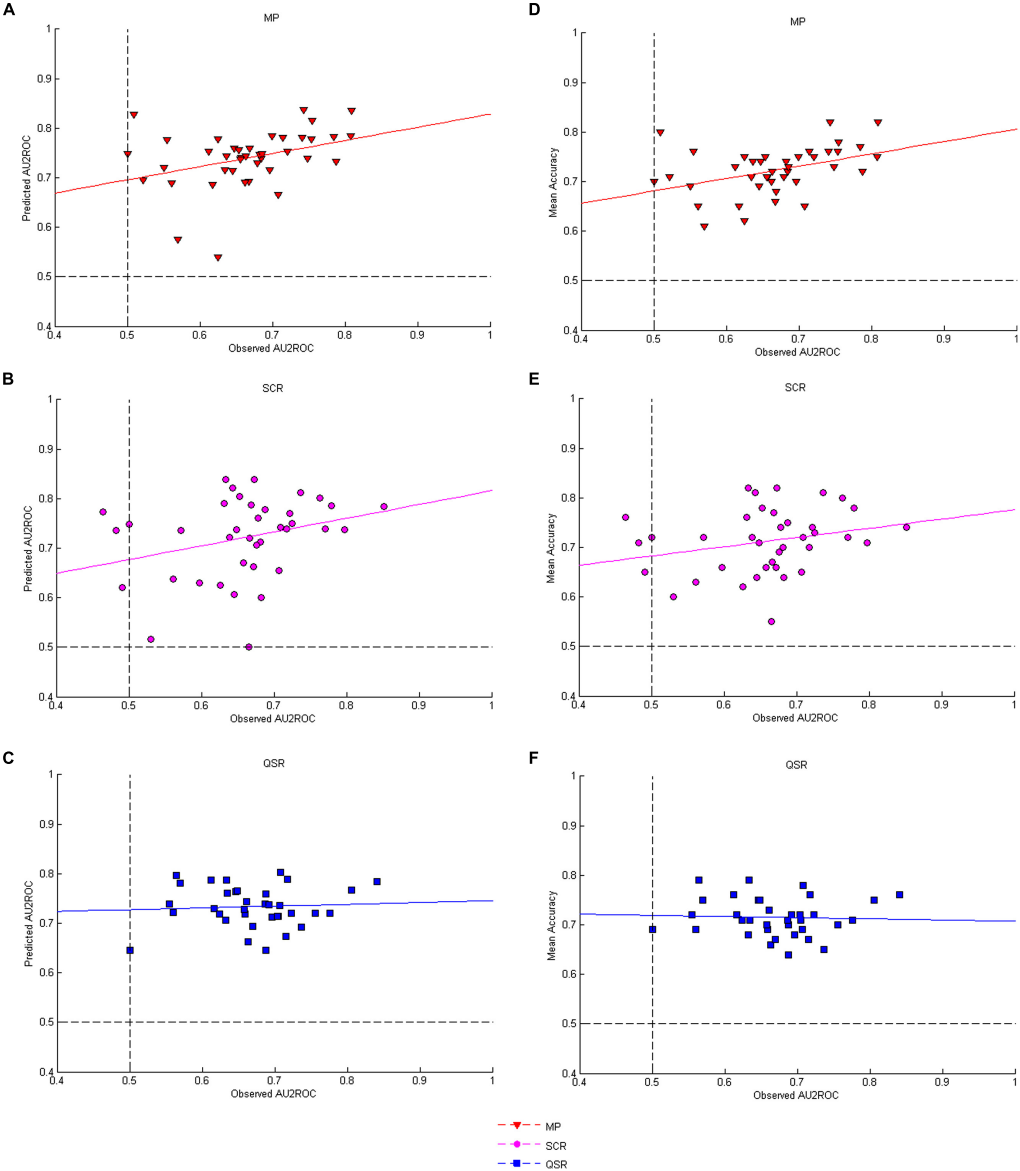
**Correlations between AU2ROC and accuracy. (A–C)** Show respectively the correlations between observed and predicted AU2ROC for the three rules; while **(D–F)** present respectively the correlations between observed AU2ROC and mean levels of accuracy for the three rules.

The second prediction states that elicited AU2ROC should be close to predicted ones. **Figures 7A–C** displays the corresponding data for each rule. The correlation between observed and predicted AU2ROC is positive and statistically significant only for the MP (**Figure 7A**, *r* = 0.36, *P* = 0.0232) while it is not statistically significant for the QSR (**Figure 7C**, *r* = 0.06, *P* = 0.7233) and only marginally significant for the SCR (**Figure 7B**, *r* = 0.29, *P* = 0.0741). The differences between these coefficients of correlations are marginally statistically significant for the MP against the QSR (Fisher-*z* = 1.31, *P* = 0.0951) but not against the SCR (Fisher-*z* = 0.33, *P* = 0.3707) and not between the SCR and the QSR (Fisher-*z* = 0.98, *P* = 0.1635).Our third prediction is that observed AU2ROC should not be greater than the predicted one. This is actually the case for 34 out of 40 subjects (85%) in the MP group, 28 out of 38 (74%) in the SCR group and 26 out of 35 (74%) in the QSR group. On average each rule provides a mean observed AU2ROC lower than the predicted one (MP: mean –0.0738, SD 0.08, *t*(40) = –5.8448, *P* < 0.0001; QSR: mean = –0.0635, SD 0.08, *t*(35) = –4.6750, *P* < 0.0001; SCR: mean = –0.0629, SD 0.10, *t*(37) = –3.8229, *P* = 0.0002).

If elicited confidence corresponds to the confidence predicted by the SDT2 model, then a good (respectively, bad) elicitation rule should be good (respectively, bad), for both the distribution of confidence and the type 2 ROC (in the sense of giving results close to those predicted by SDT). This is our fourth prediction. In other words, we should observe a positive correlation between the distances observed and predicted confidence distributions on the one hand, and the distance between observed and predicted AU2ROC on the other hand. As an indicator of distance between observed and predicted AU2ROC we use the variable *ROC_distance*. The correlations are positive and significant for the MP (with the Chi-square distance: *r* = 0.48, *P* = 0.0016; with the KS distance: *r* = 0.52, *P* = 0.0007) and the SCR (with the Chi-square distance: *r* = 0.60, *P* = 0.0001; with the KS distance: *r* = 0.36, *P* = 0.0289). In contrast, the results are less conclusive for the QSR, for which we observe a correlation between distances measured by the KS metric (*r* = 0.49, *P* = 0.0030) but not by the Chi-square metric (*r* = 0.19, *P* = 0.2828). Note that in terms of the differences of correlations we find a marginally significant difference for the MP against the QSR (Fisher-*z* = 1.59, *P* = 0.0559) and a significant difference for the SCR against the QSR (Fisher-*z* = 2.05, *P* = 0.0202) in terms of Chi-square metric.

Our last prediction concerning confidence is that we should observe a positive correlation between the mean success rate in the Type 1 task and the observed AU2ROC. We report these correlations in **Figures 7D–F**. We found that performances in Type 1 and Type 2 tasks are strongly correlated when confidence is elicited with MP (**Figure 7D**, *r* = 0.41, *P* = 0.0086). The correlation is still positive, but not significant for the SCR (**Figure 7E**, *r* = 0.25, *P* = 0.1271). More strikingly, we found no correlation between performances in Type 1 and Type 2 tasks when the QSR is used (**Figure 7F**, *r* = –0.04, *P* = 0.8004). The only significant difference of correlations is between the MP and the QSR (Fisher-*z* = 1.97, *P* = 0.0244).

Taken together, our results suggest that elicitation rules differ strongly in the kind of confidence they convey. Whereas confidence levels reported using MP are globally compatible with predicted confidence, those obtained through QSR can hardly be explained by the classical SDT model. The results concerning the SCR are less conclusive. Our conclusion at this point should be that MP seems a good rule (compared to the other ones), if one seeks to elicit confidence in line with the SDT2 model.

To summarize all these results we recall our five predictions and show for each of them whether the observed confidence matches the predicted one and how the three rules can be ranked according to their fit to the predictions.

– *Prediction 1*: an elicited confidence close to that predicted by SDT2 model;
– *Prediction 2*: an elicited AU2ROC close to the predicted one;
– *Prediction 3*: an elicited AU2ROC not greater than the predicted one;
– *Prediction 4*: the closer the elicited confidence distribution is to the predicted confidence distribution, the closer the elicited AU2ROC is to the predicted one;
– *Prediction 5*: a positive correlation between performance in perceptual task and elicited AU2ROC.

This **Table 3** clearly shows that the MP outperforms the other rules: it is the only one that leads to confidence data respecting all the predictions and it performs as the best rule for four of the five predictions. On the contrary the QSR gives the worst results with almost three predictions not matched.

**Table 3:** Summary of the performances of the three rules according to their fit to the predictions and their relative ranking for each prediction.

	MP		SCR		QSR	
	Match	Rank	Match	Rank	Match	Rank
*Prediction 1*	Yes	1	Yes	2	Yes	3
*Prediction 2*	Yes	1	Part No	2	No	3
*Prediction 3*	Yes	1	Yes	3	Yes	2
*Prediction 4*	Yes	2	Yes	1	Part No	3
*Prediction 5*	Yes	1	Yes	2	No	3

## DISCUSSION

Dienes and Seth (2010) compared three methods for measuring consciousness: verbal report, post-wagering method, and an original “no-loss gambling” procedure. They found that, in an implicit learning task (artificial grammar), the no-loss gambling method proved to be no less sensitive than the two other ones, while being immune to subjects’ attitude towards risk.

The purpose of this study was similar in spirit. Our aim was to compare different method to measure confidence. We compared three elicitation methods for retrospective confidence judgements in a perceptual task with respect to their ability to fit SDT predictions. We found that the MP (which is a fine-grained version of the no-loss gambling method) outperforms the SCR (a direct report on a numerical scale) and the QSR (a post-wagering method). These results thus show that the choice of the method for confidence measurement matters greatly, and provide support for the use of the MP in studies of confidence judgements that are based on SDT analysis.

A possible explanation for these results could be as follows. First, it is known that QSR is plagued with individuals’ risk aversion (see Schurger and Sher, 2008; Dienes and Seth, 2010; Fleming and Dolan, 2010). Furthermore, it is expressed in terms of stakes, and not in terms of probabilities or confidence levels. It might be that this simple fact requires some “translations” (from probabilities to stakes) that distort individuals’ reports. By contrast, both SCR and MP are directly expressed in terms of confidence rating, and are immune to risk aversion. Moreover, MP provides incentives to truthfully report one’s confidence, which is not the case of SCR. This might explain that MP performs better. Finally we cannot exclude that the weak performances of the QSR and the SCR are not linked to the misunderstanding by our subjects on how to use these scales.

Incidentally, if one is willing to interpret confidence as a degree of consciousness, our results can also be read as a confirmation of those of Dienes and Seth (2010) for perceptual tasks, fine-grained measurement methods, and using a different comparison criterion (proximity to the prediction of an ideal Bayesian observer).

This study presents some limitations. First we use the most basic SDT model in order to predict confidence. Even if our results are robust enough to draw some conclusion about the ability of the MP to fit SDT predictions, it could be interesting to confirm these results by using SDT in a more sophisticated way. Remaining in a static framework, one could refine the SDT model in order to take into account possible position bias and unequal variance of signals (Wickens, 2002). If we were to extend our analysis to a dynamic setting, the diffusion model seems to be a powerful tool to understand Type 1 (see Ratcliff and McKoon, 2008, for a review) and Type 2 (Ratcliff and Starns, 2009; Pleskac and Busemeyer, 2010) decisions. Unfortunately our data does not allow these two refinements of SDT. The second drawback of this study comes from our experimental design. Our analysis is based on between-subjects comparison: each individual only uses one of the three elicitation rules. As metacognitive ability is known to be very heterogeneous between subjects (Fleming et al., 2010) and as a switch of rules during an experimental session has proved to be too confusing, a proper protocol could be to ask subjects to come for several sessions, spaced out by time, with the use of a new rule at each session.

Recent studies on metacognition have mainly focussed on the measure of metacognitive ability and its variation across individuals or across tasks, using SDT analysis as a theoretical framework. In the present study we take another point of view by trying to identify which elicitation method is the most appropriate to measure confidence in line with SDT framework. Our results support the idea that the choice of elicitation rules matters and provide evidence that experiments which use SDT as a theoretical basis should elicit confidence by the MP mechanism.

## AUTHOR CONTRIBUTIONS

Sébastien Massoni and Jean-Christophe Vergnaud designed the study and collected the data. Sébastien Massoni, Thibault Gajdos and Jean-Christophe Vergnaud analyzed the data and wrote the manuscript. All authors approved the final version of the manuscript.

## Conflict of Interest Statement

The authors declare that the research was conducted in the absence of any commercial or financial relationships that could be construed as a potential conflict of interest.

## Footnotes

1 The question whether confidence ratings are appropriate measures of consciousness is hotly debated in and of itself (Sandberg et al., 2010).

2 These experiments were part of Sébastien Massoni’s Master’s thesis (see Massoni, 2009).

3 Note that all authors worked at the University of Paris 1 during the collection of the data.

4 Pilot experiments have shown that subjects get confused when asked to use different elicitation rules in the same experimental session.

5 Note that the scales can be used on a full scale basis (0–100) but we explain during the instructions that the incentives are based on half scale (50–100) except in the case of error detection. In practice levels of confidence under 50% are scarce (2.37% overall).

6 Note that some subjects never used the 50% level of confidence: respectively 9, 14, and 5 for the MP, the SCR and the QSR.

7 We do not observe such a result for the MP that is also based on a scale with a pre-filled level of confidence at 75%. We suspect that this is due to the fact that no incentive is provided in the SCR, and that this might lead subjects simply not to make the effort to change this value in many cases. Indeed we observe that 30.8% of the elicited values are on 75% with the SCR while it is only 14.2% with the MP.
